# Activity of the Carboxy-Terminal Peptide Region of the Teneurins and Its Role in Neuronal Function and Behavior in Mammals

**DOI:** 10.3389/fnins.2019.00581

**Published:** 2019-07-31

**Authors:** David W. Hogg, Mia Husić, David Wosnick, Thomas Dodsworth, Andrea L. D’Aquila, David A. Lovejoy

**Affiliations:** Department of Cell and Systems Biology, University of Toronto, Toronto, ON, Canada

**Keywords:** TCAP, teneurin, metabolism, glucose, stress, peptide evolution

## Abstract

Teneurin C-terminal associated peptides (TCAPs) are an evolutionarily ancient family of 40- to 41-residue bioactive peptides located on the extracellular end of each of the four teneurin transmembrane proteins. TCAP-1 may exist as a tethered peptide at the teneurin-1 carboxy end or as an independent peptide that is either released via post-transcriptional cleavage from its teneurin-1 pro-protein or independently expressed as its own mRNA. In neurons, soluble TCAP-1 acts as a paracrine factor to regulate cellular activity and neuroplastic interactions. *In vitro* studies indicate that, by itself, synthetic TCAP-1 promotes neuron growth and protects cells from chemical insult. *In vivo*, TCAP-1 increases hippocampal neuron spine density, reduces stress-induced behavior and ablates cocaine-seeking behaviors. Together, these studies suggest that the physiological effects of TCAP-1 are a result of an inhibition of corticotropin-releasing factor (CRF) activity leading to increased energy production. This hypothesis is supported by *in vivo* functional positron emissions tomography studies, which demonstrate that TCAP-1 significantly increases glucose uptake in rat brain. Complimentary *in vitro* studies show that enhanced glucose uptake is the result of TCAP-1-induced insertion of the glucose transporter into the neuronal plasma membrane, leading to increased glucose uptake and ATP production. Interestingly, TCAP-1-mediated glucose uptake occurs through a novel insulin-independent pathway. This review will focus on examining the role of TCAP on neuronal energy metabolism in the central nervous system.

## Introduction

The teneurins are a family of type-II transmembrane glycoproteins that are widely expressed in the central nervous system (Baumgartner et al., 1994; Levine et al., 1994). They are involved in a number of cellular processes including the regulation of synaptic adhesion and maintenance of synaptic structures (Hong et al., 2012; Mosca et al., 2012; Boucard et al., 2014; Mosca and Luo, 2014; Li et al., 2018). Initially discovered in *Drosophila*, the teneurins were first thought to be related to the tenascin proteins. Baumgartner and colleagues were in search of *Drosophila* orthologs of the vertebrate tenascins when they identified two proteins and subsequently named them tenascin-like protein major (ten-m) and tenascin-like protein accessory (ten-a) (Baumgartner and Chiquet-Ehrismann, 1993; Baumgartner et al., 1994). At the same time, Levine et al. (1994) conducted a screen for novel phosphotyrosine-containing proteins when they identified a protein with homology to the tenascin family and named it odd Oz (Odz), as the *odz* mutant embryos did not contain odd-numbered body segments. Shortly after, a ten-m homolog was identified in chicken and termed teneurin-1, due to its robust expression pattern in the nervous system (Minet et al., 1999). Since then, these proteins were found to be structurally and functionally distinct from the tenascins, and the name teneurin was adopted as the standard nomenclature to reflect their initial discovery and their neuronal expression patterns in various organisms (Tucker and Chiquet-Ehrismann, 2006). Subsequent homology studies revealed that the teneurins are present across most metazoans. Four homologs have been identified in vertebrates, whereas invertebrates contain only one copy, with the exception of insects, where two teneurin paralogs have been discovered (Baumgartner and Chiquet-Ehrismann, 1993; Baumgartner et al., 1994; Lovejoy et al., 2006; Chand et al., 2013a).

Teneurin genes encode proteins approximately 2800 amino acids in length that contain an intracellular amino terminal, a single transmembrane region and a large conserved extracellular carboxy terminal domain (Tucker et al., 2012). The four teneurin proteins (teneurin-1-4) exhibit a high degree of structural similarity to each other, as indicated by conservation of eight tenascin-type epidermal growth factor-like repeats, a cysteine rich region, five NHL domains and 26 tyrosine-aspartic acid repeats (Minet and Chiquet-Ehrismann, 2000; Kenzelmann et al., 2008; Tucker et al., 2012; Beckmann et al., 2013). Toward the distal end of the teneurin C-terminus is a short bioactive peptide sequence, termed teneurin C-terminal associated peptide (TCAP). The structure of the teneurins suggests that they evolved from a horizontal gene transfer event, which was likely mediated by a genomic internalization of a polymorphic proteinaceous toxin payload from a prokaryote donor to a choanoflagellate. This is corroborated by the widespread, yet highly conserved expression pattern of teneurins across various cell types (Drabikowski et al., 2005; Zhang et al., 2012; Chand et al., 2013b; Ferralli et al., 2018). Teneurins are most highly expressed within the central nervous system, where they form hetero- or homo-dimers to facilitate downstream signaling (Baumgartner et al., 1994; Kenzelmann et al., 2008), though their prevalence in other non-neuronal tissue types has been well-established (Baumgartner and Chiquet-Ehrismann, 1993; Baumgartner et al., 1994).

The TCAP region of the teneurins exhibits several characteristics of an independent peptide. It was first discovered during a screening for corticotropin-releasing factor (CRF)-related homologs in a rainbow trout hypothalamic cDNA library using a hamster urocortin probe (Qian et al., 2004). A clone of the C-terminal region of rainbow trout teneurin-3 was isolated and termed TCAP-3. Subsequently TCAP-1, -2 and -4 were identified, totaling four highly conserved paralogs (Wang et al., 2005). The TCAP sequence is 40–41 amino acids in length and shares a structural similarity with CRF, calcitonin and other members of the Secretin superfamily of peptides (Lovejoy et al., 2006). TCAP shows widespread genetic expression in various brain regions, with *in situ* hybridization analyses showing TCAP mRNA expression in the murine olfactory bulb, cerebellum and brainstem (Wang et al., 2005). Though TCAP and teneurin share considerable overlap in expression patterns, the TCAPs are discretely expressed in some cortical regions that lack may teneurin expression, such as regions involved in the limbic system (Zhou et al., 2003; Wang et al., 2005; Chand et al., 2013a). These studies demonstrate that TCAP and teneurin may be differentially expressed, and ultimately suggest that TCAP processing may be independent from that of the teneurins.

## Evidence of TCAP as a Small Soluble Peptide

To determine if TCAP exists as a separate gene that produces a soluble peptide independent of teneurin, the existence of TCAP-specific mRNA was investigated by Northern blot (Chand et al., 2013a). Antisense probes to the terminal exon of all four mouse teneurins labeled the four full-length teneurin transcripts, as expected. However, in the case of teneurin-1 and -3, shorter mRNA species less than 800 bases in length were readily identified. Sequence analysis of the teneurin-1 cDNA after 5′ RACE PCR-based cloning revealed a transcript of 485 bases, corresponding to the mRNA sequence encompassing two-thirds of the terminal teneurin-1 exon oriented toward the 3′ end (Chand et al., 2013a). The TCAP-1 mRNA is characterized by a short 5′ untranslated region, a peptide-encoding region of 357 bases corresponding to 118 translated residues and a 3′ untranslated region of about 74 or more bases. Within the peptide-encoding region additional postulated furin and basic residue cleavage sites were identified using the criteria suggested by Seidah and Chrétien (1997). These sites could potentially liberate smaller peptides of 107 and 41 residues. A 13 kDa band was identified by Western blot, which could correspond to either a furin cleavage product or the full-length translation product of the TCAP-1 mRNA (Chand et al., 2013a). A smaller 5 kDa band was shown in protein extracts from the vase tunicate, *Ciona intestinalis*, which could represent a soluble form of its 39-amino acid TCAP (Colacci et al., 2015; D’Aquila et al., 2017).

There is no evidence of a signal peptide in the putative translation product of the TCAP-1 mRNA, or in the equivalent TCAP regions of teneurin-2, -3, and -4 (Wang et al., 2005; Lovejoy et al., 2006; Chand et al., 2013a). Numerous bioactive peptide hormones and paracrine factors do not possess signal peptides. However, those belonging to the Secretin superfamily of ligands typically do contain such structures. Signal peptides facilitate entry into the vesicles of the secretory pathway, and peptides without this region typically remain in the cytosol. The full-length teneurins, however, do possess the hydrophobic transmembrane region that allow them to be inserted into the plasma membrane via fusion with secretory vesicles (Baumgartner et al., 1994; Levine et al., 1994). This distinction in cellular localization is apparent in the differential expression of immunoreactive teneurin-1, which is primarily found at the plasma membrane, and immunoreactive TCAP-1, which is confined to the cytosol (Chand et al., 2013a).

Taken together, these findings indicate that a soluble TCAP peptide could be liberated by direct cleavage from the teneurins, or in the case of TCAP-1 and possibly TCAP-3, transcribed as a smaller, independent mRNA that specifically encodes the TCAP region. The mechanism by which TCAP could be cleaved directly from the teneurins is not clear, though furin or prohormone convertases associated with secretory vesicles could be responsible for this (Seidah and Chrétien, 1997). However, this supposition remains mostly theoretical, as it has not been possible to confirm the existence of an endogenous soluble 40- or 41-mer TCAP.

## Identification of the Teneurin/TCAP Receptor

Although both teneurin and TCAP possess clear cellular action, the receptor mechanisms by which these effects occur were poorly understood until recently (Baumgartner et al., 1994; Levine et al., 1994; Qian et al., 2004). Early studies on the structure of TCAP showed that it contains several amino acid motifs found in peptides of comparable sizes belonging to the CRF and calcitonin families, suggesting a phylogenetic relationship between them (Lovejoy and Jahan, 2006; Lovejoy et al., 2006). Further evidence indicated that TCAP may be related to the Secretin peptide superfamily, and thus, its receptor may be part of the Secretin superfamily of GPCRs (Qian et al., 2004; Wang et al., 2005; Lovejoy and de Lannoy, 2013). However, receptor binding and activation studies showed that this is not the case, as Secretin GPCR family members had no significant interaction with TCAP-1 (Lovejoy and Barsyte-Lovejoy, unpublished observations).

In an attempt to further elucidate a putative receptor for TCAP-1, gene array studies investigating changes in gene expression of immortalized murine neuronal cells upon TCAP-1 treatment were performed. These studies showed that TCAP-1-treated cells had higher expression of dystroglycan genes than vehicle-treated cells did (Chand et al., 2012), suggesting a functional relationship between the two proteins. The dystroglycans are transmembrane proteins associated with several receptor systems, particularly those of certain growth factors, integrins and other intercellular adhesion factors (Peng et al., 2008; Lombardi et al., 2017). Interestingly, no association between dystroglycans and Secretin GPCRs has been established to date. Fluorescence studies using a FITC-labeled variant of TCAP-1 and immunolabeled dystroglycan revealed that the two are, indeed, proximal to each other on the cell membrane. Moreover, TCAP-1 treatment induces activation of a MEK-ERK signal transduction system associated with the dystroglycans (Chand et al., 2012). Despite this, no direct evidence of binding between the two has been observed to date, indicating that they likely do not form a receptor-ligand pair, and may simply be parts of a larger intercellular complex.

Further research into teneurin binding partners has revealed a putative receptor for both teneurin and TCAP. Although full-length teneurins were previously shown to homo- and heterodimerize, leading to activation of downstream signaling cascades (Kenzelmann et al., 2007), the teneurins also bind to the GPCR Latrophilin-1 (LPHN1). Together, they form a *trans-* synaptic complex with both adhesion and cell signaling properties (Silva et al., 2011; Boucard et al., 2014; Vysokov et al., 2016). The LPHNs comprise a group of three GPCRs (LPHN1-3) that were first discovered in search for a calcium-independent receptor of α-latrotoxin, the primary vertebrate toxin in black widow spider (genus *Lactrodectus* ) venom (Davletov et al., 1996). Upon their discovery, the LPHNs were initially classified as Secretin GPCRs based on the high sequence similarities of their putative hormone-binding domain (HBD) with the signature HBDs of the Secretin GPCRs (Lelianova et al., 1997). They have since been reclassified as members of the Adhesion GPCR family due to their newly discovered adhesion functions and their long extracellular domains which contain several adhesion motifs (Fredriksson et al., 2003; Nordström et al., 2009). Recent phylogenetic analyses indicate that the Adhesion GPCR family is ancestral to the Secretin GPCR family, suggesting that Secretin GPCRs inherited their HBDs from their Adhesion GPCR ancestors (Nordström et al., 2009). If this is the case, other Adhesion GPCRs may also possess additional peptide ligands that have yet to be discovered. In this respect, TCAP may act as a model system to understand peptide-receptor interactions amongst the Adhesion GPCRs.

Further to these phylogenetic studies, several recent binding studies provide clear evidence that the TCAP region is required for teneurin-LPHN interaction. LPHN1 binds LPHN1-associated synaptic surface organizer (Lasso), a splice variant of teneurin-2 comprised of the protein’s distal C-terminus, including the TCAP-2 region (Silva et al., 2011). Full-length teneurin and Lasso both exhibit a high affinity with LPHN1, suggesting that TCAP itself might likewise bind with the LPHN family as an independent ligand. Deletion of the teneurin C-terminus reduces binding of teneurin with the extracellular domains of LPHN1 (Silva et al., 2011). Additionally, recent structural studies indicate that the teneurin extracellular domain forms a barrel-like structure from which the TCAP-containing C-terminus protrudes (Jackson et al., 2018; Li et al., 2018). This conformation may make the TCAP portion of teneurin accessible to potential interacting partners, such as LPHN1, on adjacent cells and allow it to interact with said partners as either an active site of the full teneurin protein or independently as a cleavable peptide. Moreover, an interaction may occur directly between TCAP and LPHN1 at the LPHN1 HBD, as this is the region of peptide binding in Secretin GPCRs. It is also the most highly conserved region among the three LPHN isoforms and is involved in binding with other LPHN ligands such as α-latrotoxin (Holz and Habener, 1998; Krasnoperov et al., 1999). Recent studies showed that a transgenically expressed TCAP-1 construct can be immunopreciptated with a transgenically expressed HBD-containing fragment of LPHN1, indicating that an interaction does occur between these two proteins (Husić et al., 2018). In addition, over-expression of LPHN1 in HEK293 cells results in increased uptake of TCAP-1 and subsequent cytoskeletal reorganization consistent with what has been observed in other cell types upon TCAP-1 treatment, further indicating a functional interaction between these two proteins (Al Chawaf et al., 2007a; Chand et al., 2012; Husić et al., 2018).

## Bioactivity of TCAP-1

Numerous studies have indicated potent bioactivity for synthetic TCAP-1. Treatment of Gn11 cells with multiple doses of TCAP-1 showed that a low dose (1 nM) leads to an increase in intracellular cAMP levels whereas a high dose (100 nM) decreases cAMP (Wang et al., 2005). Immortalized murine hypothalamic cells also display higher survivability under stress conditions when treated with TCAP-1, where TCAP-1 treatment led to increased total cell number and decreased necrotic cell number after 48 h in cells grown under high pH conditions (Trubiani et al., 2007). Likewise, cells exposed to peroxide exhibited decreased cell death when treated with TCAP-1, pointing to a role for the peptide in neuroprotection. In addition to this, TCAP-1 may also have actions in neuroplasticity, as it has potent effects on cytoskeletal dynamics. Treatment with TCAP-1 increases β-actin and β-tubulin in murine immortalized hypothalamic cells and increases neurite outgrowth in a hippocampal cell line (Al Chawaf et al., 2007a; Tan et al., 2012). It also induces filamentous actin polymerization in a variety of cell lines through activation of a dystroglycan-associated MEK-ERK downstream signaling cascade (Chand et al., 2012; Husić et al., 2018).

These effects were further elucidated in an *in vivo* study utilizing intracerebroventricular injection of TCAP-1 under unstressed and restraint conditions. Under unstressed conditions, TCAP-1 decreased dendritic branching while under stressed conditions it increased dendritic branching (Al Chawaf, 2008). Although the mechanism is still unknown, these results provide evidence that TCAP-1 plays a role in neuroplasticity.

**FIGURE 1:**
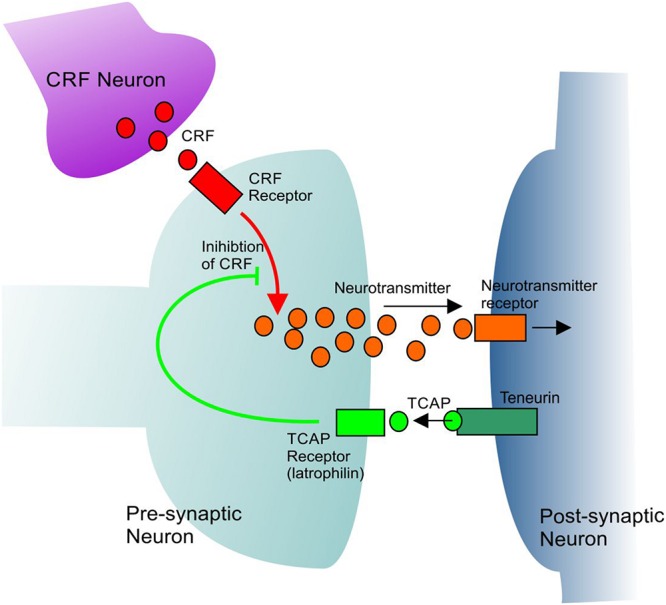
A general model showing the mechanism of CRF inhibition by TCAP. TCAP acts upon a specific receptor to inhibit the intracellular signal transmission of CRF. See text for further discussion.

Immunohistochemical analysis has revealed that immunoreactive TCAP-1 is present within the limbic system, particularly in the areas associated with regulation of the behavioral stress response, such as the pyramidal layer of the hypothalamus and the basolateral nucleus of the amygdala (Tan et al., 2012). Neurons in these regions are morphologically plastic and can change in response to stimuli such as stress and learning (Tan et al., 2009, 2011; Chen et al., 2013). The effects of TCAP-1 on stress-related behavior have been observed using several methodologies including the acoustic startle response (ASR), an indicator of anxiety and a test of reflexive fear response (Rotzinger et al., 2010). Through these studies, two major bioactive attributes of TCAP-1 have been identified: its neuromodulatory effects and its regulation of the CRF-induced stress response. In one such study, rats were separated based on their baseline ASR response prior to any treatment, and subsequently treated intracerebroventricularly with TCAP-1 (Wang et al., 2005). Rats with a low baseline ASR response exhibited an increase of in response upon TCAP-1 treatment. In contrast, rats with a high baseline ASR showed an attenuated response after treatment with TCAP-1. TCAP-1 was also shown to induce long-term attenuation of the stress response, as rats had a 50% reduction in ASR up to 15 days after TCAP-1 treatment (Wang et al., 2005). Moreover, pre-treatment with TCAP-1 is able to modulate CRF-induced stress responses in several behavioral paradigms including ASR, elevated plus maze and open field tests (Al Chawaf et al., 2007b; Tan et al., 2008; Rotzinger et al., 2010). CRF-induced cocaine reinstatement is also reduced in rats given TCAP-1 pre-treatment (Kupferschmidt et al., 2011; Erb et al., 2014). These studies highlight the role TCAP-1 has in regulating CRF-associated behaviors related to anxiety and depression in rodent models (Figure 1).

## Role of Energy Metabolism by TCAP

In both animal and cell models, synthetic TCAP-1 activates several processes that necessitate increased energy production. This includes protection of neurons against alkaline chemical insults and cell death (Trubiani et al., 2007), stimulation of neurite outgrowth, reorganization of cytoskeletal elements in neurons (Al Chawaf et al., 2007a; Tan et al., 2011; Chand et al., 2012), and modulation of stress-related behaviors (Wang et al., 2005; Al Chawaf et al., 2007b; Tan et al., 2008). These actions are energetically costly, indicating that TCAP-1 may also stimulate energy production to maintain them, as cellular supply of ATP must meet cellular energy demand.

Glucose is the preferred energy substrate in brain, and a steady supply is critical for neuronal function; however, neurons have a limited capacity to store glucose intracellularly. Therefore, a TCAP-1-mediated increase in intracellular glucose would indicate that TCAP-1 can also stimulate cellular energy metabolism. In a study using functional positron emission tomography, a single subcutaneous injection of TCAP-1 induced a significant increase in ^18^F-deoxyglucose uptake into the brain 3 days post-treatment (Hogg et al., 2018). Glucose uptake was highest in the frontal cortex and subcortical regions, although it occurred throughout the cortical regions. In addition, a single injection of TCAP-1 decreased whole animal blood glucose by 35–40%, with a concomitant decrease in serum insulin and an increase in serum glucagon. This pattern mimics the effect of insulin on blood glucose and glucagon, demonstrating that TCAP-1 alters whole animal glucose metabolism. Similar results were obtained in Goto-Kakizaki rats, a type-II diabetic insulin-insensitive pathological model, suggesting that TCAP-1-stimulated glucose uptake is independent of the insulin system.

In confirmation of these studies, TCAP-1 also increases glucose uptake in a hypothalamic neuron cell model. In these cells, TCAP-1 increased uptake of [^3^H-]2-deoxyglucose, a non-hydrolysable form of glucose, by 50% following 60 and 90 min of treatment. This profile differed from that of insulin, which induced uptake at 30 min post-treatment, further indicating that TCAP-1 regulates glucose uptake independently of insulin. This was confirmed when insulin- and TCAP-1-mediated glucose uptake were assessed in the presence and absence of a depolarizing stimulus. Insulin-mediated glucose uptake requires membrane depolarization for glucose transporter (GLUT) insertion into the plasma membrane (Uemura and Greenlee, 2006). Unlike insulin, TCAP-1-mediated glucose uptake occurrs in the absence of membrane depolarization events (Hogg et al., 2018). The timeline and depolarization-independent nature of TCAP-1-induced glucose uptake indicates that TCAP-1 activates a signaling mechanism distinct from that of insulin-mediated glucose uptake.

Glucose uptake into neurons occurs by faciltated diffusion through GLUTs, and is dependent on the plasma membrane expression of GLUTs and the diffusion gradient of glucose into the cell. The actions of TCAP-1 are consistent with this mechanism. TCAP-1 increases GLUT3 transport to the plasma membrane of a hypothalamic neuronal model by 37.5% within 1 h, where the increase is maintained for up to 3 h post-treatment (Hogg et al., 2018). In addition, TCAP-1 increases GLUT3 immunoreactivity by ∼250% in the growth cones of extending neurites 1–2 h post-treatment. Moreover, TCAP-1 does not significantly increase membrane expression of GLUT1 or GLUT4, indicating that TCAP-1-induced glucose uptake likely occurs specifically through a GLUT3-mediated process. GLUT3 is the primary glucose transporter in brain, whereas the GLUT1 transporters are typically located in endothelial cells of the blood-brain barrier, and the GLUT4 transporters are highly expressed in skeletal muscle and adipose tissue and known to be insulin-dependent (Shepherd et al., 1992; Haber et al., 1993; Choeiri et al., 2002; Airley and Mobasheri, 2007).

Increases in intracellular glucose importation stimulate cellular energy metabolism. TCAP-1 significantly increases intracellular ATP turnover in a dose-dependent manner in an immortalized neuronal cell line, demonstrating that TCAP-1-mediated glucose uptake does, indeed, stimulate neuronal energy metabolism (Hogg et al., 2018). This increase in intracellular ATP turnover is likely the result of stimulation of aerobic energy-producing pathways, as TCAP-1 also decreases cellular lactate concentrations. Cellular pyruvate concentrations likewise decreased, indicating the metabolic pathway favors producing pyruvate for oxidative energy production. Previous studies have demonstrated that TCAP-1 can significantly increase catalase, superoxide dismutase and the superoxide dismutase copper chaperone (Trubiani et al., 2007), reducing intracellular reactive oxygen species. If TCAP-1 is increasing mitochondrial activity, there would be a consequential increase of reactive oxygen species production, thus the TCAP-1 system may have evolved a mechanism to compensate this. Taken together, these data demonstrate that TCAP-1 is a functional component of the teneurin protein that regulates glucose uptake and neuronal energetics in the rodent brain.

## Conclusion

Teneurin C-terminal associated peptide represents a bioactive region of the teneurin proteins that may act as a tethered ligand or a distinct peptide that is either cleaved from the full-length teneurin protein or expressed independently (Chand et al., 2013a). TCAP-1 has several energetically favorable functions, and increases uptake of glucose into the rodent brain (Hogg et al., 2018). This indicates that it can act to increase energy availability, allowing for its other implicated functions to take place. The exact mechanism by which TCAP-1 acts is yet to be elucidated; however, TCAP-1-mediated glucose uptake appears to be independent of insulin. Although further studies are required to tease out the precise signaling cascade that TCAP-1 induces to facilitate increased neuronal glucose uptake via GLUT3, the current studies presented in this review reveal a novel and essential function of this peptide family, further supporting TCAP as a critical stress-response regulator.

## Author Contributions

DH: analysis of energy metabolism and description of key studies. MH: interpretation of receptor interaction and associated experiments, review and editing of the manuscript. DW: overview of aerobic studies on TCAP and associated literature. TD: molecular biology of TCAP receptor interactions and associated literature. AD’A: development of key experiments associated with TCAP action. DL: supervision of research program and overview of the manuscript.

## Conflict of Interest Statement

DL is a co-founder of Protagenic Therapeutics, Inc., a commercial entity that may have interests in the findings of these studies. The remaining authors declare that the research was conducted in the absence of any commercial or financial relationships that could be construed as a potential conflict of interest.
